# Zidovudine (AZT) and Hepatic Lipid Accumulation: Implication of Inflammation, Oxidative and Endoplasmic Reticulum Stress Mediators

**DOI:** 10.1371/journal.pone.0076850

**Published:** 2013-10-11

**Authors:** Atrayee Banerjee, Mohamed A. Abdelmegeed, Sehwan Jang, Byoung-Joon Song

**Affiliations:** Laboratory of Membrane Biochemistry and Biophysics, National Institute on Alcohol Abuse and Alcoholism, Bethesda, Maryland, United States of America; UAE University, Faculty of Medicine & Health Sciences, United Arab Emirates

## Abstract

The clinical effectiveness of Zidovudine (AZT) is constrained due to its side-effects including hepatic steatosis and toxicity. However, the mechanism(s) of hepatic lipid accumulation in AZT-treated individuals is unknown. We hypothesized that AZT-mediated oxidative and endoplasmic reticulum (ER) stress may play a role in the AZT-induced hepatic lipid accumulation. AZT treatment of C57BL/6J female mice (400 mg/day/kg body weight, i.p.) for 10 consecutive days significantly increased hepatic triglyceride levels and inflammation. Markers of oxidative stress such as protein oxidation, nitration, glycation and lipid peroxidation were significantly higher in the AZT-treated mice compared to vehicle controls. Further, the levels of ER stress marker proteins like GRP78, p-PERK, and p-eIF2α were significantly elevated in AZT-treated mice. The level of nuclear SREBP-1c, a transcription factor involved in fat synthesis, was increased while significantly decreased protein levels of phospho-acetyl-CoA carboxylase, phospho-AMP kinase and PPARα as well as inactivation of 3-keto-acyl-CoA thiolase in the mitochondrial fatty acid β-oxidation pathway were observed in AZT-exposed mice compared to those in control animals. Collectively, these data suggest that elevated oxidative and ER stress plays a key role, at least partially, in lipid accumulation, inflammation and hepatotoxicity in AZT-treated mice.

## Introduction

Highly active anti-retroviral therapy (HAART) has significantly decreased the rate of mortality and morbidity in AIDS patients [1], [2], [3]. Zidovudine (3-azido-3′-deoxythymidine or AZT), a nucleoside reverse transcriptase inhibitor, was the first HAART drug approved by the U.S. Food and Drug Administration for treatment of patients with AIDS, and is still the backbone of the large proportion of HAART treated patients in the developing world where majority of HIV infected patients resides [4]. However, the clinical effectiveness of AZT is constrained due to its adverse side effects [5]; the most common of which is hepatotoxicity [6], [7]. In fact, the incidence of HAART-related severe hepatotoxicity has been reported to be approximately 10% of the HIV infected people under medication, and life threatening events occur at a rate of 2.6 per 100 person years [8], [9]. Further, a significantly increased number of HIV-infected individuals treated with AZT died from hepatotoxicity resulting from lipid dysregulation, steatosis, steatohepatitis, hepatomegaly and abnormal liver function [10], [11], [12], [13], [14].

AZT, a potent inhibitor of the replication of human immunodeficiency virus (HIV), has been known to induce oxidative stress in the muscle, liver, heart and immortalized blood brain barrier endothelial cells [15], [16], [17], [18]. This has been attributed to the damage and depletion of mitochondrial DNA, probably by the AZT-induced inhibition of DNA polymerase γ [19], [20]. In addition, oxidative stress has been known to play a role in the fat accumulation and inflammation in alcoholic and non-alcoholic fatty liver diseases [21], [22]. For instance, recent studies from this laboratory have suggested that increased oxidative stress promotes oxidative/nitrative modification of mitochondrial enzymes involved in the fat oxidation pathway, leading to significantly elevated hepatosteatosis [21], [22], [23]. In addition to oxidative stress, endoplasmic reticulum (ER) stress also plays a contributing role in hepatic lipid accumulation. Prolonged exposure to reactive oxygen species (ROS) and/or nitrogen species (RNS) often leads to changes in protein structure, function and activity. In fact, various chaperone proteins in the ER were oxidatively modified under increased oxidative/nitrative stress, as shown [24]. Under conditions of sustained oxidative stress, the ER cannot eliminate many, if not all, of the misfolded proteins, and hence the cells experience the ER stress response. Recent studies have also indicated that ER stress induces an abnormal signaling associated with lipid homeostasis [25], [26]. However, the mechanisms of fat accumulation and hepatotoxicity observed in the AZT-exposed patients are poorly understood. Based on the aforementioned studies, we aimed to investigate the potential mechanism(s) of AZT-mediated fat accumulation in mice by evaluating: (1) the levels of inflammation, oxidative and ER stress marker proteins; (2) the post-translational protein modifications; and (3) the levels of critical proteins involved in fat synthesis and mitochondrial fat degradation.

## Materials and Methods

### Materials

All chemicals used in this study including AZT were highest grades and obtained from Sigma Chemical (St. Louis, MO, USA). The specific antibodies for CYP2E1, 3-NT and iNOS were purchased from Abcam (Cambridge, MA). Anti-AGE antibody was from Fitzgerald Biotechnology (Acton, MA) while anti-CYP3A and anti-CYP4A were gifts from Dr. James P. Hardwick. Anti-PPARα and anti-OPN antibodies were from Santa Cruz Biotechnology (Santa Cruz, CA). All other antibodies against GRP78, p-PERK, p-eIF2α, IRE1α, p-AMPK, p-ACC, SREBP-1, ATP synthase, tubulin, histone H3 and β-actin were obtained from Cell Signaling Technology (Boston, MA).

### Animal Treatment and Sample Collection

Age-matched C57BL/6 female mice were randomly assigned to two groups – saline control (n = 6) and AZT-treated mice (n = 8). Female mice were used for the experiments, as they have been reported to have higher AZT-mediated toxicity, as compared to their male counterparts [27]. To study the mechanisms of hepatic fat accumulation, we used a pharmacological dose of AZT, as described [27]. The animals were injected intraperitoneally (i.p.) with saline or AZT (400 mg/day/kg body weight for 10 consecutive days and sacrificed 24 h after the last injection, as described [28]). Throughout the AZT treatment, the animals were kept in a 12 h light-dark cycle with food and water available *ad libitum*. Following euthanasia by asphyxiation, the whole liver harvested from each mouse was divided into two parts. A small portion of the largest lobe of each liver was fixed in 10% neutral buffered formalin, while the remaining tissue was snap frozen and stored at −80°C for further analysis. All animal procedures were carried out in accordance with the NIH guidelines and all efforts were made to minimize any suffering. The protocol for AZT treatment and humane care were approved by the Institutional Animal Care and Use Committee of National Institute on Alcohol Abuse and Alcoholism. For histopathology, 4 µm-thick paraffin-embedded liver sections were cut and stained with H&E for bright-field light microscopy. To assess the degree of inflammation, the number of inflammatory foci per five high power fields was quantified from the H& E stained liver sections.

### Sample Processing

To prepare whole liver homogenates, liver tissues were homogenized in an extraction buffer (50 mM Tris-HCl, pH 7.5, 1 mM EDTA and 1% CHAPS), pre-equilibrated with nitrogen gas to remove the dissolved oxygen, as previously described [21]. To isolate nuclear, cytoplasmic and mitochondrial fractions, liver tissues were homogenized on ice in 5 volume of STE buffer (250 mM sucrose, 50 mM Tris-HCl, pH 7.5, 1 mM EDTA, with protease and phosphatase inhibitors). The buffers, used in this study, were saturated with nitrogen gas for 1 h to remove the dissolved oxygen [29]. The homogenates were then subjected to differential centrifugation, followed by washing, to separate the nuclear, cytoplasmic and mitochondrial fractions [22], [29]. The concentration of the proteins was determined using the BioRad protein assay kit, following the manufacturer’s protocol as described previously [22], [29].

### Hepatic Triglyceride Assay

Liver tissues (50 mg wet weight) homogenized in 5% Triton X-100 solution, were heated in 80–100°C water bath for 2–5 min to solubilize the triglycerides. The samples were then centrifuged at 10,000×*g* for 10 min, and the collected supernatant was used to determine the triglyceride level following the manufacturer’s protocol (BioVision Research products, Mountain View, CA).

### Determination of Alanine Aminotransferase (ALT), MDA+HAE, and Hydrogen Peroxide (H_2_O_2_) Concentrations

The level of ALT was measured in the plasma of each mouse using the clinical IDEXX Vet Test chemistry analyzer system (IDEXX Laboratories, West Brook, ME, USA). The amount of MDA+HAE was measured using the commercially available kit from Oxford Biomedical research Inc. (Oxford, MI) by following the manufacturer’s protocol. Mitochondrial protein (100 µg/assay) from each animal was used for the experiments. H_2_O_2_ produced from total liver homogenates was determined during 30-min incubation by using the Amplex Red hydrogen peroxide assay kit from Molecular Probes (Eugene, OR) by following the manufactures protocol [30], [31].

### Immunoblot Analysis

Different cellular fractions (30–70 µg protein) were separated by 10 or 12% polyacrylamide gel electrophoresis and electrophoretically transferred to PVDF membranes. Upon completion of the transfer, the membranes were blocked for 1–3 h with 5% milk or bovine serum albumin (BSA) in Tris-HCl buffered saline containing 0.01% Tween 20 (TBS-T). The membranes were then probed with specific primary antibodies in 5% milk or BSA in TBS-T overnight at 4°C. After washing the remaining primary antibodies three times at 10-min intervals, the membranes were incubated with the respective secondary antibodies for 1 h at room temperature. Final visualization was carried out with the enhanced chemiluminescence kit (Bio-Rad, Hercules, CA) with Kodak X-OMAT film and their densities quantified using Image J software.

### Protein Oxidation and Activity Measurements

The carbonylated protein levels were determined with the Oxyblot protein oxidation detection kit following the manufacturer’s protocol (Millipore, Billerica, MA, USA). The activity of 3-ketoacyl-CoA thiolase (thiolase) was determined by the absorbance change at 303 nm following the disappearance of the Mg^2+^-enolate complex of acetoacetyl-CoA, as previously described [22], [29].

### Statistical Analysis

Data represent results from at least three separate measurements. Values are expressed as means ± SEM, unless otherwise indicated. Statistical analysis was performed using the Student’s t-test. Statistical significance was set up at p<0.05. Other methods not specifically described here were the same as previously described [22], [29], [31], [32].

## Results

### Increased Hepatic Fat Accumulation, Inflammation, and Toxicity in AZT-exposed Mice

**Figure 1:**
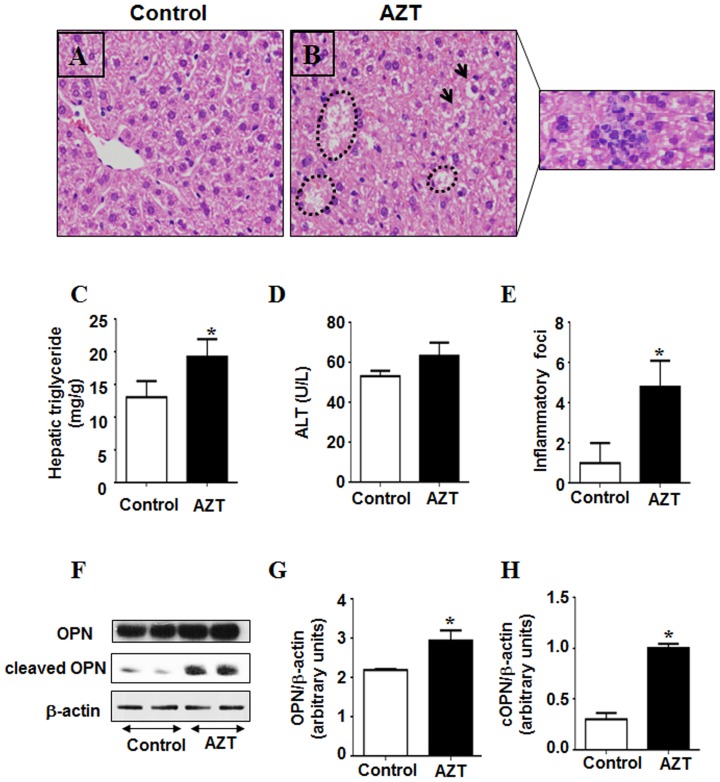
AZT-mediated hepatic fat accumulation, inflammation, and injury. (A, B) Representative photomicrographs of H&E stained liver sections from the indicated mouse livers are presented: (A) vehicle-control; (B) AZT–treated mice (outside inset: inflammatory foci as they are scattered). Accumulated fat are indicated by circular lines and arrows. (C) Hepatic triglyceride levels, (D) Serum ALT levels, and (E) inflammation foci (per 5 high power fields) are presented for the different groups. F) Equal amounts of total liver homogenates (50 µg/lane) from different groups were used to evaluate the amounts of full-length OPN, cleaved (active) OPN and β-actin protein (as a loading control). (G, H) Densities of full-length OPN and cleaved OPN (cOPN) protein bands were normalized to that of the β-actin. *Significantly different from the control group (n = 6/group). Data indicate mean±SE, p<0.05. All experiments have been conducted three times.

Histological analysis with H & E staining revealed that mice exposed to AZT for 10 consecutive days exhibited mild or moderate hepatic lipid accumulation along with clear presence of inflammatory foci and some scattered necrosis, compared to the controls (Figs. 1A, B). Consistently, the histological data corroborated with mild to moderate but significantly higher hepatic triglyceride (TG) levels in the AZT-exposed mice, when compared to the vehicle control (Fig. 1C). Plasma ALT levels were not significantly different between the groups, although the ALT level of the AZT-treated animals seems to be higher than the vehicle control (Fig. 1D).

In addition to steatosis, hepatic inflammatory foci were significantly increased in AZT-exposed animals compared to the control (Fig. 1E). This was further confirmed by the increase in the proinflammatory marker osteopontin (OPN). The levels of both full-length and cleaved OPN (cOPN) were significantly increased in the livers of mice treated with AZT compared to controls (Figs. 1F–H). These results indicate that AZT treated animals had significantly higher fat accumulation and inflammation than their control counterparts.

### Increased Oxidative Stress in AZT-treated Mice

**Figure 2:**
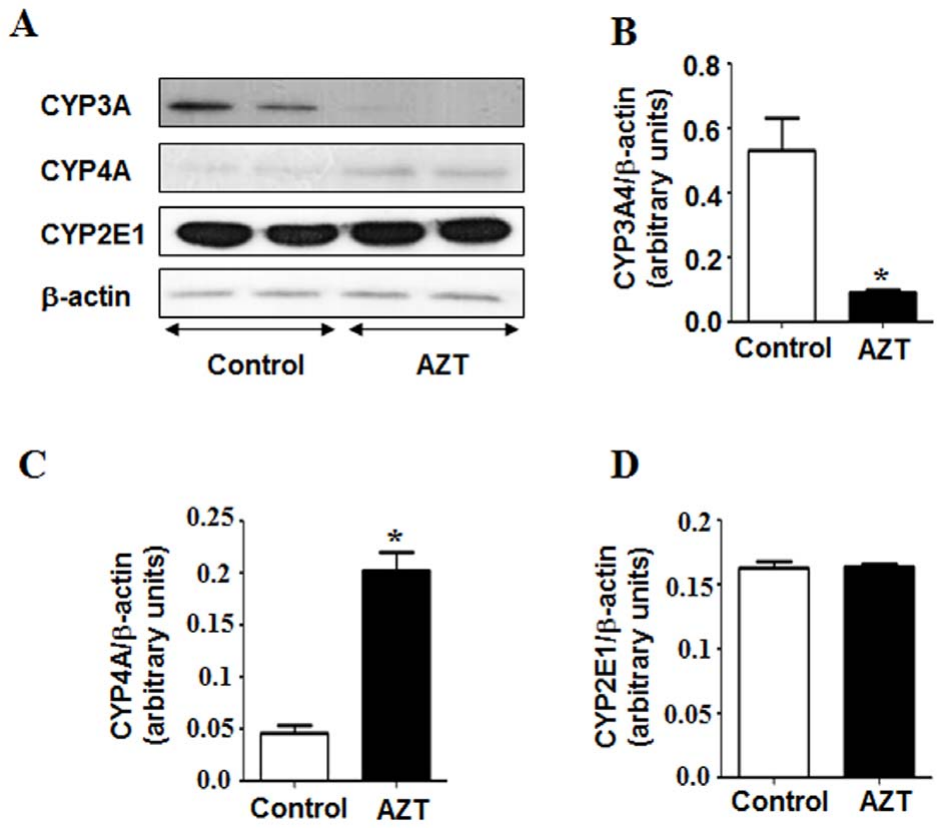
Effect of AZT on the levels of CYP3A4, CYP4A and CYP2E1 proteins. (A) Equal amounts of whole liver homogenates (50 µg/lane) from both groups were used to evaluate the levels of CYP3A4, CYP4A and CYP2E1 (upper, middle and lower panel), respectively. (B, C, D) The densities of each immunoreactive band were normalized to that of β-actin as the loading control. *Significantly different from the control group. Data indicate mean±SE, p<0.05. All experiments have been conducted three times.

The cytochrome P450 enzyme CYP3A4 is one of the major enzymes responsible for the metabolism and degradation of many therapeutic agents. Previous reports indicated that the expression of CYP3A4 protein is suppressed in the presence of oxidative stress and fatty liver [33], [34]. Consistent with these reports, we observed that AZT-exposed animals had significantly lower levels of CYP3A4 protein than those of their vehicle controls (Figs. 2A, B).

**Figure 3:**
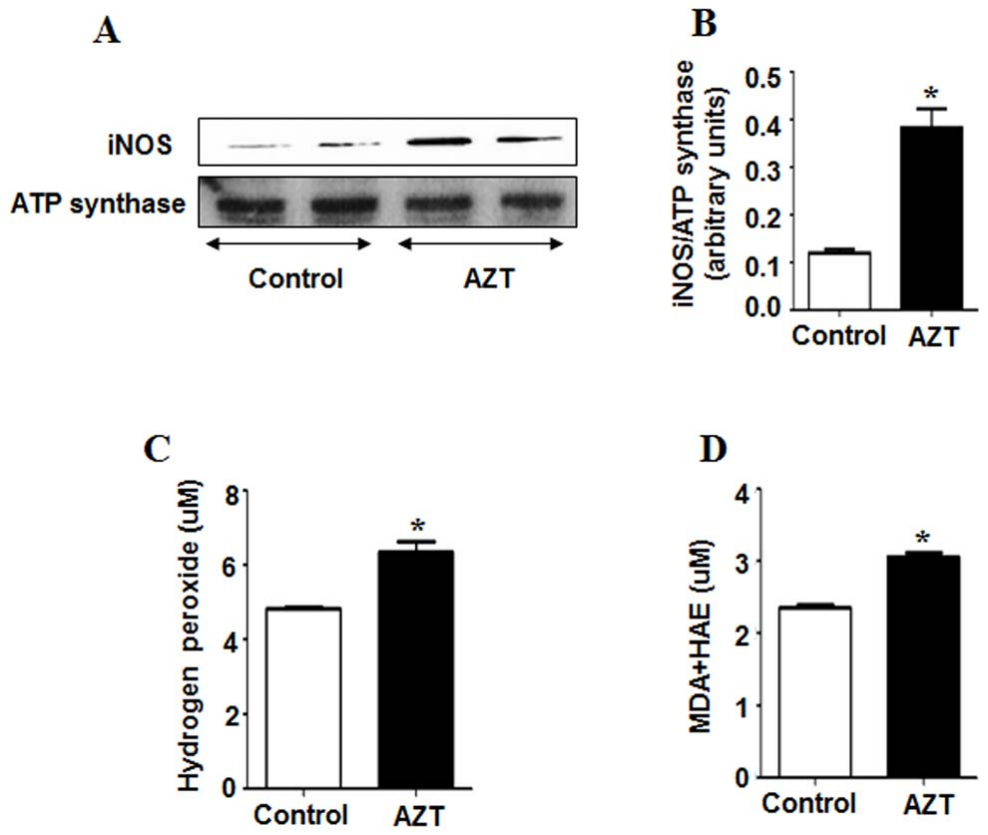
AZT-mediated increments of oxidative stress markers. (A) Equal amounts of mitochondrial proteins (50 µg/lane) were used to evaluate the levels of mitochondrial iNOS (upper panel) and ATP synthase (lower panel) from both groups. (B) The densities of iNOS bands were normalized to that of ATP synthase, the loading control. (C) The rate of production of hydrogen peroxide and (D) the level of MDA+HAE as a marker of lipid peroxidation was measured as described in the Materials and Methods. *Significantly different from the control group. Data indicate mean±SE, p<0.05. All experiments have been conducted three times.

Other cytochrome P450 enzymes like CYP4A and CYP2E1 are known to produce ROS during their catalytic cycles of fatty acid metabolism [35]. Because both CYP4A and CYP2E1 are producers of ROS, we also determined their expressed levels in our model. Interestingly, the expression of CYP4A was significantly higher in the AZT-exposed mice than that of the controls, implicating higher oxidative stress in AZT-treated animals, whereas the levels of CYP2E1 protein were similar in both groups (Figs. 2A, C, and D). In addition, we measured the levels of inducible isoform of NOS (iNOS, mitochondrial), lipid peroxidation and H_2_O_2_ production. AZT treatment significantly elevated the expression of iNOS (Figs. 3A, B). As illustrated (Figs. 3C, D), the rate of H_2_O_2_ production and the MDA+HAE levels significantly increased in the AZT-treated animals, as compared to the controls, indicating that AZT likely elevated oxidative and nitrative stress in mice.

**Figure 4:**
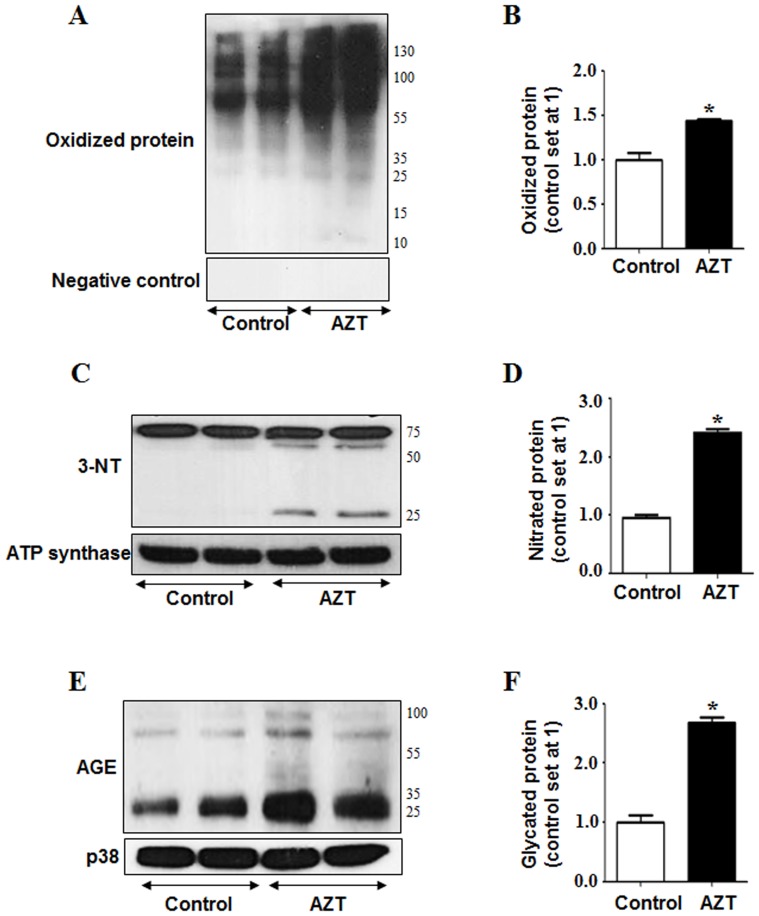
AZT-mediated protein modifications. (A) Equal amounts of mitochondrial proteins (50 µg/lane) from the different groups were used to evaluate protein oxidation (upper panel) using Oxyblot analysis or negative control (lower panel). (B) Densities of mitochondrial oxidized proteins were normalized with that of the controls set at 1. (C) Equal amounts of mitochondrial proteins were used to evaluate protein nitration as reflected by 3-NT bands (upper panel) and ATP synthase loading control (lower panel) from different groups. (D) The densities of mitochondrial 3-NT bands were normalized to that of ATP synthase and plotted as the value of controls being 1. (E) Total liver homogenates (50 µg/lane) were used to evaluate the levels of AGE (upper panel) and p38 (lower panel) as a loading control. (F) The densities of AGE bands were normalized to that of p38 and plotted as the value of controls being 1. *Significantly different from the control group. Data indicate mean±SE, p<0.05. All experiments have been conducted three times.

Recent reports from our laboratory demonstrated that increased protein oxidation observed at early time points contributes to mitochondrial dysfunction long before full-blown liver disease caused by many hepatotoxic agents and ischemia reperfusion injury [29], [31]. Since AZT-treated mice exhibited higher oxidative/nitrative stress, as evident by higher levels of CYP4A isozyme, lipid peroxidation, H_2_O_2_ and iNOS, we analyzed the levels of oxidized and nitrated proteins, respectively. AZT treatment significantly increased the amounts of oxidized mitochondrial proteins compared to the control counterparts (Figs. 4A top panel, B). The specificity of the oxidized protein bands has been confirmed using a 1X derivatization control solution, where no bands were detected (Fig. 4A, bottom panel). In addition, elevated nitrative stress, reflected by nitrated (3-NT) proteins, can modify many cellular macromolecules and inactivate various target proteins [32]. The levels of nitrated mitochondrial proteins reflected by 3-NT immunoreactivity were significantly elevated in the AZT-treated mice compared to the corresponding controls (Figs. 4C, D). Taken together, these results reveal that AZT significantly increased oxidative/nitrative stress. Elevated oxidative stress is also known to increase the levels of advanced glycation end products (AGE). As shown in Figs. 4E and F, AZT treatment significantly elevated the levels of AGE, while p38 levels, used as a loading control, were similar in all samples.

### Elevated ER Stress and Altered Fat Metabolism in AZT-exposed Mice

**Figure 5:**
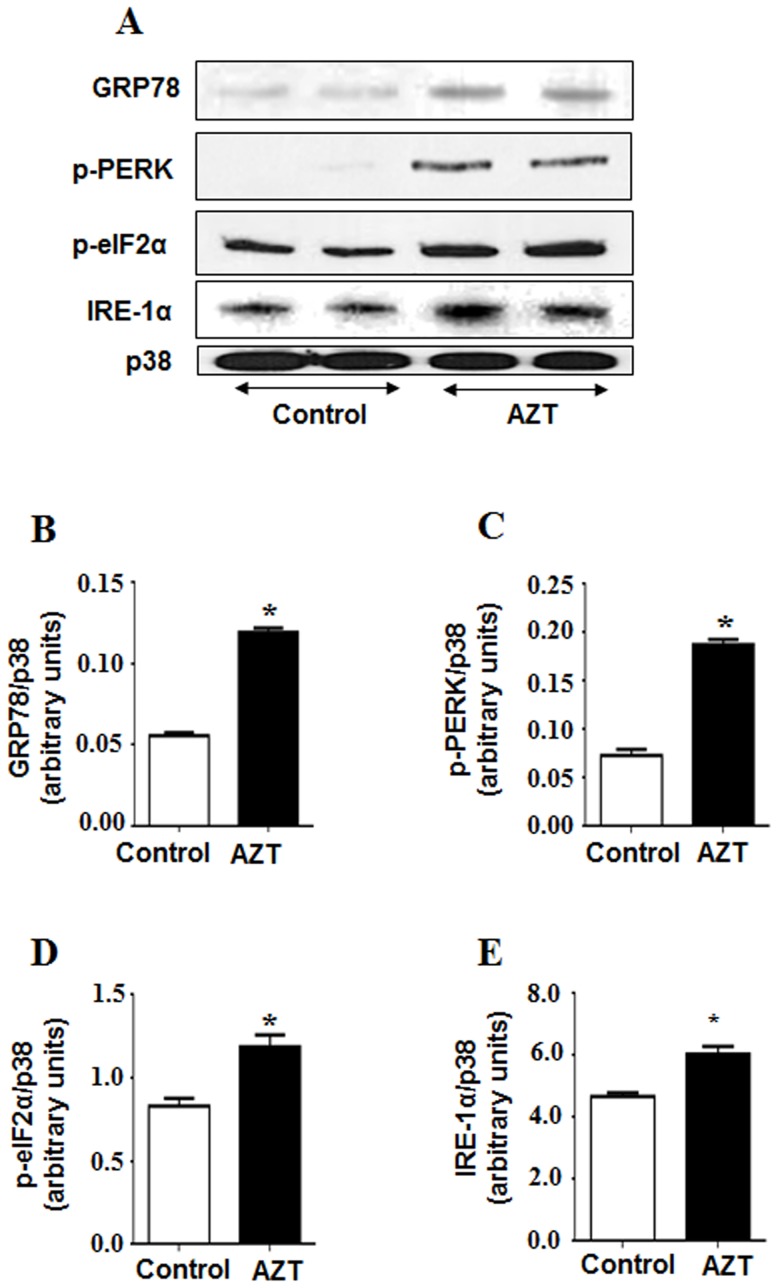
AZT-induced increases in the ER stress markers. (A) Equal amounts of total liver homogenates (50 µg/lane) from different groups were used to evaluate the levels of GRP78, p-PERK, p-eIf 2α, IRE-1α and p38 (as a loading control). (B, C, D, E) The densities of each protein were normalized to that of p38 and presented. *Significantly different from the control group. Data indicate mean±SE, p<0.05. All experiments have been conducted three times.

To test whether AZT-mediated hepatic injury is promoted through increased ER stress, we analyzed the hepatic levels of ER stress markers such as molecular chaperone glucose-regulated protein 78 (GRP78) and the phosphorylation status of its downstream targets including protein kinase-like endoplasmic reticulum kinase (PERK) and the eukaryotic translation initiation factor-2α (eIF2α) (Figs. 5 A–E). Compared to the control, AZT treatment significantly promoted ER stress as indicated by the increased amounts of GRP78, p-PERK, p-eIF2α and inositol-requiring and ER-to-nucleus signaling protein 1α (IRE1α).

**Figure 6:**
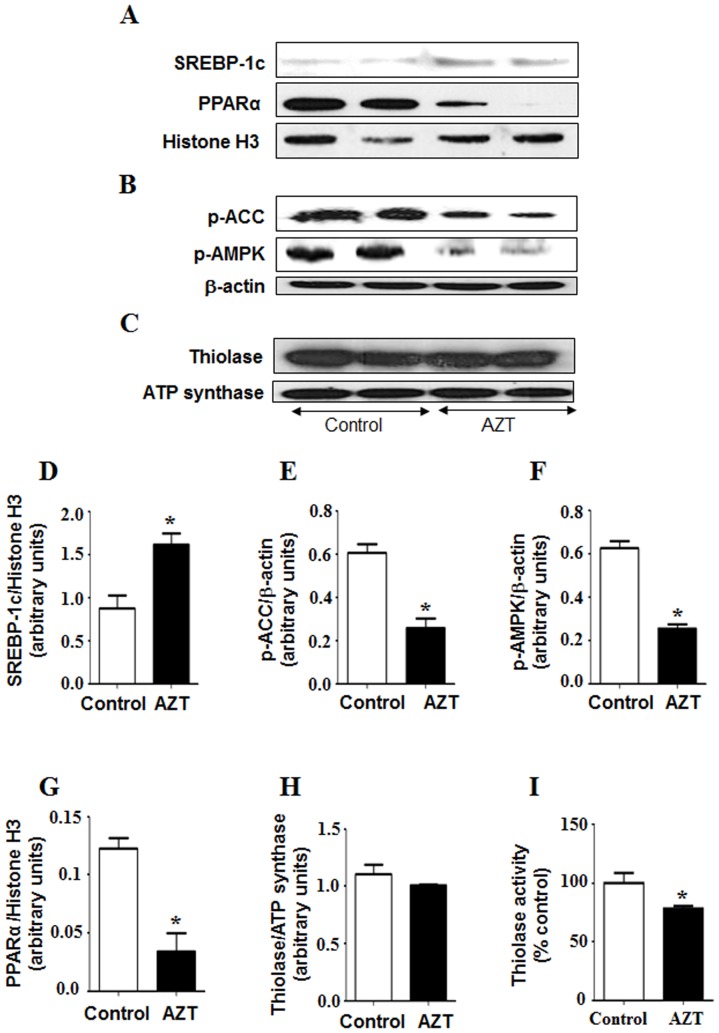
AZT-induced alterations of key proteins involved in fat metabolism. Equal amounts of nuclear, total liver homogenates and mitochondria from different groups were used to measure the levels of (A) n-SREBP-1, PPARα and Histone H3 (as loading control for nuclear proteins) (B) p-ACC, p-AMPK and β-actin (as a loading control for total liver homogenates), and (C) thiolase and ATP synthase (as a loading control for mitochondrial proteins), respectively. (D–H) The density of each protein was normalized to that of the indicated loading control, respectively. (I) Thiolase activity was measured as described in the Materials and Methods. *Significantly different from the control group. Data indicate mean±SE, p<0.05. All experiments have been conducted three times.

To determine whether alteration in fatty acid metabolism might also play a role in the hepatic fat accumulation, we evaluated the expression of some critical proteins involved in this process. The level of sterol regulatory element binding protein 1c (SREBP-1c), a transcription factor involved in fat synthesis, was significantly elevated in the AZT-exposed animals (Figs. 6A, D). In addition, the levels of phospho-acetyl-CoA carboxylase (p-ACC) and phosphorylated AMP activated kinase (p-AMPK), major enzymes in controlling fatty acid biosynthesis, were significantly decreased in AZT-exposed mice compared to the controls (Fig. 6B, E, and F respectively). AZT exposure decreased the levels of peroxisome proliferator activator receptor α (PPARα) (Figs. 6A, G), a transcription factor involved in fat transport, degradation and export of free fatty acids. Furthermore, the activities of 3-keto-acyl-CoA thiolase in control and AZT-exposed mice were 142.4 units and 111.0 units, respectively, suggesting that AZT treatment significantly (approximately 22%) suppressed the activity of thiolase, the last enzyme in the fatty acid β-oxidation pathway (Fig. 6C, H, and I) despite similar protein levels in both groups. Together, these results indicate that hepatic fat accumulation in AZT-exposed mice likely result also from both increased fat synthesis and suppression of the fat degradation pathway.

## Discussion

Antiviral drug regimens, especially those employing NRTIs have been closely associated with altered fat metabolism and fat distribution in humans [36], [37], [38]. However, the mechanism(s) by which NRTIs cause fat accumulation are poorly understood. AZT, the most commonly prescribed drug among the NRTIs, is frequently associated with various side effects including hepatotoxicity and hyperlipidemia. Previous studies have reported significant histological alterations such as fatty liver, vacuolar degeneration and microvesicular fat accumulation in the liver of rats treated with 1 mg/ml of AZT in drinking water for up to 90 days, and the lesions were found to be progressively greater with the longer duration of AZT treatment [13]. However, the potential mediators of fat accumulation in AZT-exposed rats were not studied. Herein, we examined whether AZT causes hepatotoxic and steatotic effects in a relatively shorter duration of treatment when following the established regimen [28]. In our model, we observed that mice treated with AZT for 10 days exhibited mild but significantly higher levels of hepatic TG and fat accumulation. It is possible that more prolonged treatment with AZT would produce greater levels of steatosis; however, this model was suitable to characterize some of the immediate potential steatotic mediators. This was accompanied with significant increase in inflammation, implicating acute liver injury (Fig. 1). Despite the fact that there was no significant difference in the ALT levels between the groups, this lack of significant increase in ALT is not uncommon in the presence of hepatic injury and is in concordance with several previous studies where underlying hepatic disorders were observed in the absence of elevated serum transaminase levels [21], [39], [40].

De La Asuncion *et al*. reported that AZT mediated toxicity could impair mitochondrial function and cellular metabolism by producing ROS with increased oxidative stress in liver and muscle [15], [16]. To correlate with the increased H_2_O_2_ production we observed with AZT in this study, we also determined the levels microsomal proteins CYP4A and CYP2E1, since these proteins could be potential sources for ROS production in various liver disease models. In *Cyp2e1* null-mice, increased expression of CYP4A has been observed during lipid accumulation in nonalcoholic fatty liver (NAFLD) and steatohepatitis (NASH) caused by high fat diets (HFD) [41] or methione-choline-deficient diet [42]. Induction of CYP4A is suggested to prevent lipotoxicity from free fatty acids (FFA), leading to a negative feedback loop which increases uncoupling of the P450 catalytic cycle, resulting in increased ROS production [41]. Thus the increased CYP4A along with elevated levels of iNOS are likely responsible for the increased production of ROS and reactive nitrogen species (RNS) including NO. Increased production of ROS and RNS ultimately leads to peroxynitrite formation, as reflected by increased 3-NT proteins in the AZT-treated mice, similar to the results in AZT-exposed cardiomyocytes [17]. Consistently, we observed significantly higher levels of lipid peroxides, oxidized and nitrated proteins in the livers of the AZT-treated mice than their corresponding control counterparts. To our knowledge, this is the first report of AZT-induced oxidative and nitrative protein modifications. Oxidative/nitrative stress has been reported to cause various post-translational modifications of Cys-residues of cellular proteins [23], [29], [32], which negatively affect physiological functions of some essential proteins, prior to the observation of full-blown liver disease [29]. In addition, AZT-treated mice showed significantly higher levels of hepatic AGEs, which can potently trigger ROS production, as demonstrated in experimental models of NASH [21], [43], [44], as compared to the vehicle-treated control, further confirming higher oxidative stress in AZT-treated animals. Further, Gao et al. [45] have shown that production of ROS in AZT-exposed primary cardiomyocytes could be attenuated by using resveratrol, a plant-derived anti-oxidant. Thus all these results indicate that AZT treatment increases hepatic oxidative/nitrative stress and promotes various protein modifications possibly including oxidative inactivation of 3-keto-acyl-CoA thiolase involved in the fatty acid degradation pathway. These events likely contribute to increased fat accumulation and hepatocellular damage (Fig. 4), as reported in rats exposed to binge ethanol [22] or ischemia-reperfusion [29].

It is well-established that alcoholic and nonalcoholic fatty liver diseases are accompanied by significant activation of ER stress response [46], [47]. In animal models, HFD induces the expression of the chaperone GRP78 and phosphorylates its downstream targets PERK and eIF2α, the key components of the ER stress pathway involved in suppressing protein translation [48], [49]. Consistent with these reports, our results showed that AZT significantly increased the levels of GRP78 and phosphorylation of PERK and eIF2α (Fig. 5). Further, PERK-mediated phosphorylation of eIF2α was shown to increase inflammation [50], [51]. Similarly, we observed significantly increased levels of a proinflammatory cytokine OPN (both full-length and cleaved active forms) in AZT-treated mice compared to those in the control, indicating higher inflammation. These results demonstrate that AZT significantly increased hepatic ER stress and inflammatory responses, as observed (Fig. 1). However, the temporal relationship among oxidative stress, ER stress, and inflammation will require further investigation, which might reflect a vicious cycle developed among the three risk factors.

Cholesterol and lipid synthesis are primarily controlled by the master transcription factor SREBP-1 [52], [53]. Previous studies have shown that HIV protease inhibitors like ritonavir and indinavir significantly increased the mature forms of nuclear SREBPs, leading to an increase in intracellular lipids and foam cell formation [54]. Feeding with an ethanol liquid diet also significantly elevated the level of active (mature) form of hepatic SREBP-1, accompanied by increased expression of lipogenic enzymes and hepatic TG [53], [55]. The activation of SREBP-1 has been reported to be partly regulated through the inhibition of AMPK, a key regulator of lipid metabolism [56]. AMPK is also known to phosphorylate and inactivate ACC, a rate limiting enzyme for fatty acid biosynthesis [57]. From both *in vitro* and *in vivo* studies, ethanol treatment inhibits AMPK activity followed by increased levels of mature SREBP-1 and ACC activity [58], [59]. Similar to these reports, we observed elevated levels of TG and hepatic lipid accumulation accompanied by decrease in active p-AMPK and increased amounts of mature SREBP-1c and ACC proteins in the AZT-exposed group (Fig. 6). These results indicate that AZT likely increased fatty acid synthesis.

In addition, decreased fatty acid oxidation can lead to increased hepatic lipid accumulation. Nuclear hormone receptor PPARα is a key transcription factor involved in the transport and oxidation of FFAs. Previous studies from this laboratory demonstrated that PPARα-null mice were more sensitive to NAFLD and NASH induced by HFD and fasting [21], [23]. Similar to these reports, we observed that AZT significantly decreased the levels of PPARα compared to the vehicle-treated controls. PPARα is also known to regulate the enzymes like cytochrome P450 CYP4A. However, during hepatic steatosis and steatohepatitis, CYP4A (a member of fatty acid ω-hydroxylase family) can be induced (Fig. 2) even with the down-regulated PPARα (Fig. 6), as recently described [41]. Further, the increase in hepatic fat accumulation can also be attributed to the inhibition of peroxisomal and mitochondrial fatty acid β-oxidation pathways, as evidenced by the decrease in the activity of mitochondrial thiolase (Fig. 6). The decrease in 3-keto-acyl-CoA thiolase activity could be due to oxidative modification of its active site Cys residues under increased oxidative stress [22]. Taken together, these results indicate that AZT treatment altered some critical proteins in fat metabolism, leading to elevated lipid accumulation.

**Figure 7:**
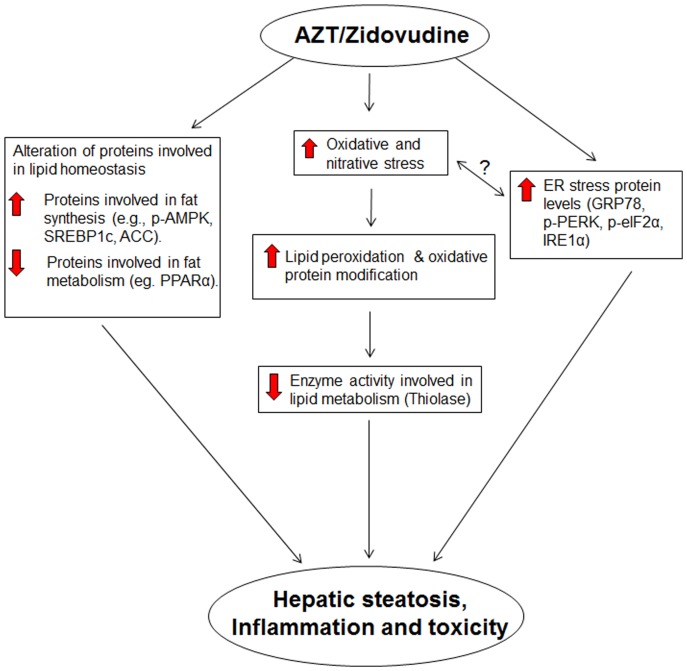
Schematic diagram describing potential mediators of AZT-mediated steatosis and injury. AZT-induced hepatic fat accumulation and injury is mediated through increased oxidative and nitrative stress, lipid peroxidation, protein modifications and inflammation.

In summary, our current results implicate that AZT treatment leads to lipid accumulation, inflammation, and toxicity in the liver likely through increased oxidative and ER stress. Our results also show that increased lipid accumulation is due to altered fatty acid metabolism, as indicated by increased levels of nuclear SREBP-1c along with significantly decreased levels of inactive phospho-ACC and active phospho-AMPK, PPARα and thiolase (Fig. 7). Collectively, these results provide the first evidence that oxidative and ER stresses play a role, at least partially, in hepatic lipid accumulation and inflammation in AZT-exposed animals. The direct relationship between oxidative stress, ER stress and the development of fatty liver will warrant further investigation using anti-oxidants and/or ER stress inhibitors to dissect the temporal occurrence of each event.
